# Position-specific physical and technical demands during the 2019 COPA América Football tournament

**DOI:** 10.17159/2078-516X/2021/v33i1a11955

**Published:** 2021-12-20

**Authors:** A Kubayi

**Affiliations:** Department of Sport, Rehabilitation and Dental Sciences, Faculty of Science, Tshwane University of Technology, Pretoria, South Africa

**Keywords:** distance, sprinting, match, tactics, passes

## Abstract

**Background:**

Despite a substantial body of literature on the physical and technical performance characteristics of football players in Asian and European tournaments, research on South American football players is scarce.

**Objectives:**

The purpose of the study was to examine the physical and technical characteristics of football players according to specific playing positions at the 2019 COPA América tournament.

**Methods:**

A total of 180 match observations from 13 games were monitored using the InStat tracking system. Players were grouped into the following five playing positions: central defenders (n = 45), wide defenders (n = 46), central midfielders (n = 50), wide midfielders (n = 17), and forwards (n = 22).

**Results:**

Descriptive statistics (means ± standard deviations) and the one-way analysis of variance were used to analyse the data. Findings showed that the total distance covered by central midfielders (10 553 ± 763 m) was significantly (*p* < 0.05) higher than that of central defenders (9226 ± 720 m; effect size (*d)* = 1.79), wide defenders (9929 ± 633 m; *d* = 0.89) and forwards (9383 ± 820 m; *d* = 1.45). Wide midfielders (214 ± 170 m), wide defenders (152 ± 199 m) and forwards (138 ± 94 m) covered greater distances sprinting than central defenders (67 ± 42 m; *d* = 1.19) and central midfielders (91 ± 66 m; *d* = 0.95). Concerning technical variables, central midfielders played significantly more passes compared to players in other playing positions (*p* < 0.05). In relation to crossing, wide defenders completed significantly more crosses than players in other positions (*p* < 0.05).

**Conclusion:**

These findings have direct implications for tailoring tactics so players can meet the physical and technical demands of the game.

The optimal physical preparation of professional football players has become an integral part of the game, particularly due to the increased physical demands during a match.^[1]^ Technological advances have led to sophisticated systems capable of recording and processing the physical and technical data of all players during a game now being used in elite club settings. In addition, monitoring work rate profiles of players during tournaments is now possible through the use of high-quality cameras and modern computer software.^[1]^ These computerised, semi-automated image recognition systems provide real-time movement information of all players during match play. This means more advanced analytical evaluations of the specific elements of an individual player’s match performance can be generated on a large sample of players.^[2]^

Regardless of the different methods used to assess footballers’ activity profiles,^[3]^ players generally cover a total distance of 8–12 km during a match, with the vast majority at low intensity, such as walking and jogging.^[4]^ Players’ physical demands based on match-related playing position have been well described in the literature.^[2–5]^ For example, Bradley et al.^[3]^ found that wide and central midfielders covered a greater total distance than fullbacks, attackers, and central defenders in European competitions. Previous research has also shown that attackers cover greater high-intensity running distances compared to players in other positions.^[4]^ While it has been reported that total distance is not a discriminatory indicator for successful performance,^[5]^ high-intensity activities are important for match outcomes as they contribute to team success.^[2]^ High-intensity efforts allow players to quickly reach optimum speeds and are a crucial component of match-deciding moments in football.^[3]^

While previous research using semi-automatic video analysis provided useful information about physical indicators of football players, little attention has been given to information on the technical demands of players.^[5]^ Research has shown technical and tactical abilities are considered important for success in soccer.^[6]^ In their study, Dellal et al.^[5]^ reported full backs and central defenders won most passes and heading duels in the English Premier League and Spanish La Liga. It was further reported that wide defenders, central midfielders, and wide midfielders recorded the lowest percentage of accurate passes. In a related study, Ermidis et al.^[7]^ examined the technical demands of professional football players in the 2015 Asian Cup. Findings showed that central midfielders performed more passes than central defenders, external midfielders, and forwards. In addition, forwards had more aerial duels than fullbacks, central midfielders, and external midfielders.

In the past decade, several studies have investigated the physical and technical performance attributes of football players in Asian and European domestic leagues and continental competitions.^[2–5]^ In contrast, there is limited research on the match-play performance indicators of South American football players. Such research is important because the playing styles of South American national football teams are usually characterised by skill and flair, whereas the European games are characterised by a direct style of play and ball possession.^[8]^ Therefore, given the disparity in playing styles across these two continents, a better understanding of players’ physical and technical attributes in South America would be helpful for football coaches and scientists to devise match-winning tactics. In addition, sports scientists, coaches and fitness trainers will be able to design research, game strategy and fitness training programmes based on individual player characteristics.^[9]^ The purpose of this study was to examine the physical and technical characteristics of football players according to specific playing positions at the 2019 COPA América tournament.

## Methods

### Participants

The sample consisted of 180 match observations from 13 games during the 2019 Confederación Sudamericana de Fútbol (CONMEBOL) COPA América football competition. Players in this study were categorised into the following five position groups: central defenders (n = 45), wide defenders (n = 46), central midfielders (n = 50), wide midfielders (n = 17), and forwards (n = 22).^[10,11]^ Only players who finished the full 90 minutes were included in the analysis. Goalkeepers, players who were replaced, and those who were substitutes were excluded from the analysis. This study received ethical clearance from the Faculty of Science Ethics Committee of Tshwane University of Technology.

### Physical and technical indicators

The players’ match performances were captured using the InStat tracking system. Their physical movement activities included walking, jogging, running, high-speed running, sprinting and completing the total distance. The InStat system has been shown to be highly accurate with levels of absolute and relative reliability, typical errors (from 0.019 to 0.036) and total errors (from 0.020 to 0.037).^[12]^ Technical indicators consisted of total passes; percentage of accurate passes; shots; crosses; dribbles; total air challenges; air challenges won; tackles; percentage of tackles won; lost balls; and fouls committed. The operational definitions of the physical and technical variables are provided in Table 1.^[11–15]^

### Statistical analysis

Data were reported as means ± standard deviations. Data normality was checked using the Kolmogorov–Smirnov test. A one-way analysis of variance (ANOVA) was used to compare the physical and technical indicators of soccer players across five playing positions. The Tukey HSD post-hoc analysis was further performed if the *F*-ratio was significant at *p*≤0.05. Cohen’s effect size (*d)* was applied to examine the magnitude of the differences in the mean scores of the studied variables. Effect size was classified as trivial (<0.20); small (0.20–0.59); moderate (0.60–1.19); large (1.20–2.00); and very large (>2.00).^[16]^ All statistical analyses were conducted using the IBM SPSS version 26, Armonk, NY: IBM Corp.

## Results

Table 2 shows the physical and technical indicators of football players according to playing position. Regarding physical performance, central midfielders covered greater total distances compared to players in other positions. Post-hoc comparisons using the Tukey HSD test indicated the mean total distance for central midfielders was significantly (*p* < 0.05) different to that of central defenders (*d* = 1.79), wide defenders (*d* = 0.89) and forwards (*d* = 1.45). However, there was no significant (*p*>0.05) difference between the total distance covered by central midfielders and wide midfielders (*d* =0.20). Contrary to other positions, central defenders recorded the lowest overall total distance. Further, descriptive statistics indicated wide midfielders, wide defenders and forwards covered the greater distances while sprinting compared to central defenders (*d* = 1.19) and central midfielders (*d* = 0.95).

Concerning the technical variables, central midfielders played more passes compared to players in all the other playing positions. Post-hoc analysis revealed that the mean value of passes for central midfielders was significantly (*p* < 0.05) different from that of the wide midfielders (*d* = 1.10) and forwards (*d* = 1.90). Descriptive statistics indicated that the central defenders and central midfielders had a greater percentage of passing accuracy compared to wide defenders, wide midfielders, and forwards. The post-hoc analysis showed the mean percentage of passing accuracy for central midfielders was significantly (*p* < 0.05) different from that of the wide midfielders (*d* = 1.09) and forwards (*d* = 1.28). Central defenders did not differ significantly (*p* > 0.05) from either the wide defenders (*d* = 0.42) or central midfielders (*d =* 0.11). In relation to crossing, wide defenders completed more crosses than players in other positions. Interestingly, the mean crosses score for wide defenders was significantly (*p* < 0.05) different from that of wide midfielders (*d* = 0.62).

When considering the loss of the ball, the results indicated that the forwards lost the ball on significantly more occasions than players in any of the other playing positions (*p* < 0.05). The post-hoc comparison showed the mean value of lost balls for forwards was significantly (*p* < 0.05) different from that of the central defenders (*d* =1.89), wide defenders (*d =* 1.25), central midfielders (d= 1.27) and wide midfielders (*d* = 0.60). The descriptive statistics indicated that while forwards were involved in more aerial challenges, central defenders won more aerial challenges than all other positions. In addition, forwards completed more dribbles than players in other playing positions. Post-hoc analysis revealed that the average number of dribbles for forwards was significantly (*p* < 0.05) different from that of central defenders (*d =* 1.92), wide defenders (*d* = 1.02) and central midfielders (*d* = 0.95). Finally, central midfielders, forwards and wide midfielders committed more fouls than central defenders and wide defenders. However, the post-hoc comparison indicated that central midfielders were significantly (*p* < 0.05) different from the central defenders (*d* = 0.81).

## Discussion

The current study aimed to investigate the physical and technical demands across individual positional roles during the 2019 CONMEBOL COPA América football championship. The current observations showed the average total distance covered per match by South American players, irrespective of playing position, was 9 903 m, ranging from 7 534 m to 11 899 m. These results are lower than those reported by Kubayi^[4]^, who found that the overall distance covered by players during the UEFA European football championship matches was 10 350 m, ranging from 8 446 m to 12 982 m. Since these studies use the same observational methods to classify movements of the players during the game, it is proposed that matches at the COPA América tournament may require players to cover less distance compared to the more physically demanding European Football championships. This finding is comparable with previous research, which reported that English Premier League players covered significantly greater distances during matches than the South American players.^[17]^ The differences in work-rate between the two groups may be a direct consequence of the tactical restrictions placed on the players because of the different types of competition. In Europe, the game is traditionally played at a fast pace and requires individuals to perform a high level of activity to receive the ball from a teammate or to pressurise opponents to regain ball possession. From a South American perspective, the tactical emphasis may be placed on producing quick decisive passing movements when an opportunity is presented or created. Such tactical restraints reduce the need for players to be highly active when trying to regain possession of the ball and thereby may reduce their total distance covered.^[17]^ While it was not an aim of this paper to make physical comparisons between international competitions, future research may consider exploring the different style of play across the two continents.

When the various positional roles were compared, midfielders covered a greater distance per match compared to players in other positions. This finding corroborates previous findings which indicate midfielders cover greater distances per match due to their linking role in the team,^[8]^ both with or without the ball, thus highlighting their indefatigable role associated with covering long distances during a match.^[4]^ The finding in which wide defenders covered a larger total distance than central defenders and forwards is noteworthy. The tactical roles of wide defenders have evolved in modern soccer, as these players are required to operate in both attacking and defensive contexts.^[2]^ This dual role can subject the player to greater overall efforts and may require them to have higher fitness levels than players in the role previously.^[5]^ It is therefore important for soccer coaches and scientists to apply the concept of individual positional differences when conditioning players and to be linked to the playing style for players to meet the demands of the game.

The present results showed that the players who sprinted for greater distances were wide midfielders, wide defenders, and forwards. A high demand for these movement patterns in attacking players (i.e. wide midfielders and forwards) is possibly as a result of the need to complete explosive moves away from defending players to create space or capitalise on goal scoring opportunities.^[2]^ Furthermore, previous studies have demonstrated the high-intensity activities (e.g. sprinting) completed by wide defenders may be due to playing tactics and dynamic formations,^[10]^ whereby wide defenders contribute in the offensive phase of play; however, if there is turnover of possession, they must return quickly, especially through sprinting, to their defensive role.^[2]^

The central midfielders covered the fewest metres in sprinting. This finding may reflect their tactical duties in which they must link the defence and offence which requires continuous running, rather than only explosive movements. It should also be noted, that depending on the team tactics, players in this position may not be required to find attacking positions, but rather contribute defensively and therefore, they reduce the amount of high-speed running required.^[18]^ Nevertheless, the current study indicates variations in high-intensity movement patterns for different positions and therefore coaches should consider the development of training programmes according to individual playing roles (e.g. wide defenders). In line with previous studies,^[5]^ small-sided games, which incorporate repeated sprint activities and intermittent exercises, may be developed to optimise the performance of soccer players.

The present study indicated midfielders played significantly more passes compared to players in other positions. This result is expected, considering that teams build their attacks through the midfield Central midfielders may be involved in transitions from defence to attack or attack to defence more than other players.^[7]^ Forwards, by contrast, had the lowest number of passes, which may be attributed to the specific role of forwards as they often have their backs to the goal during link-up play.^[5]^ Consequently, forwards may have limited options to pass the ball in contrast to central midfielders. Another finding was that the central defenders had a greater percentage of accurate passes compared to players in other positions. This finding may reflect the tactics of teams to use the backline more effectively when adopting a possession-based style of play, such as playing out from the back.^[10]^

In relation to crossing the ball, wide players delivered more crosses than any other player. This finding is not surprising given these players operate in wider positions on the pitch, which means that one of their main roles is to cross the ball into the box to create a goal-scoring opportunity. When considering the differences between these wide playing positions, it was interesting to note that wide defenders attempted significantly more crosses with moderate effect per match compared to wide midfielders. This demonstrates the evolving nature of the wide defender role in the modern game and the need to also to be effective in the attacking phases of play. It could also be inferred that South American teams are adopting formations/tactics which allow the wide midfielders to tuck inside during an attacking phase of play and encouraging wide defenders to move to the final third of the field to put a cross in the box. While exploring this style of play was not an aim of the current investigation, future research may consider the use of this overlapping tactic and how it may contribute to successful team performance.

There was a significant difference in the number of dribbling actions completed across the playing positions, with forwards completing significantly more dribbling actions compared to the midfielders. This finding is inconsistent with that of Taylor et al.^[19]^ who found midfielders in Europe performed the highest number of dribbles during a game. One of the roles for forwards is to penetrate the opposition defence to create goal-scoring opportunities. One method to do this may be via dribbling at the defenders. A characteristic of the South American style of play is skill and flair, of which dribbling can be classified.^[8]^ In contrast, central defenders completed the fewest number of dribbles per match, which may be attributed to the fact they tend to take fewer risks when in possession of the ball, as a mistake (i.e. of losing the ball to the opposition) can lead to a goal.^[7]^ This perspective is also supported by the fact that defenders had the fewest number of lost balls per match. Conversely, attacking players had the greatest number of lost balls. This is probably because attacking players take more risks when in possession of the ball as they attempt to penetrate the opposition’s defence, but they are also performing actions far from their own goal.^[20]^ This result could reflect the position of the forwards, as they are generally positioned in a dense zone on the pitch and are often outnumbered by the defenders. In addition to the numerical disadvantage, forwards usually play with their backs to the goal and receive the ball with a defender marking them. Thus, it is generally difficult to control the ball and turn to the goal, whereas it is easier for the defender to intercept the ball when they are positioned at the front of the game.^[20]^

In terms of the aerial element of the game, forwards and central defenders had the highest number of aerial challenges per match compared to that of any other position. However, when comparing their success rate, central defenders won a greater proportion of aerial duels compared to the forwards. This supports Taylor et al’s^[19]^ finding where defensive players have the highest number of clearances compared to that of attacking players. However, the low number of aerial duels won among forwards may be attributed to the fact they are often competing against the strongest defensive players on the opposition during aerial challenges.^[7,19]^ Consistent with previous research,^[7]^ coaches should consider developing training sessions which incorporate heading exercises to respond to the demands of playing in both defensive and attacking positions. In addition, attacking players were found to have committed more fouls than defensive players. This may reflect the modern game, whereby players are encouraged to start defending from the opponent’s half by pressing to delay or interfere with the attack.^[5]^

### Limitations and future research

Despite the novel information on physical and technical requirements across playing positions in South America investigated in this study, there are several limitations that should be considered. Firstly, the study only analysed an available sub-sample (n = 13) of data from the 2019 COPA América. This may limit the generalisation of the findings to the whole tournament. Secondly, while the current study has incorporated peer-reviewed published operational definitions of players’ movements, comparisons with other studies are limited due to different observational methods and classifications used across studies. Finally, other variables, such as environmental factors, match outcome, the quality of the opposition, and the importance of the games were not assessed in the current study. Therefore, future research may consider including a larger sample size and the effect of situational variables (e.g., match outcomes, playing formations, playing style, etc.) on the physical and technical performance of soccer players.

## Conclusion

The purpose of this study was to examine the physical and technical demands of football players according to positional roles at the 2019 COPA América tournament. The findings indicated that there are specific position variations in physical and technical demands. The knowledge gained from this study may allow for a greater understanding of the physical and technical requirements for football players in the Copa América and may have direct implications for devising match tactics. Thus, football coaches should develop training programmes according to individual playing position, so players are prepared to meet their physical and tactical roles during a match.

## Figures and Tables

**Table 1:** Operational definitions of physical and technical indicators

Variable	Definition
**Physical indicators**	
Walking	Distance covered at a speed of 0–7 km.h^−1^ during a match.
Jogging	Distance covered at a speed of >7–14.5 km.h^−1^ during a match.
Running	Distance covered at a speed of >14.5–20 km.h^−1^ during a match.
High-speed running	Distance covered at a speed of >20–25 km.h^−1^ during a match.
Sprinting	Distance covered at a speed of >25 km.h^−1^ during a match.
Total distance	All distances covered during a match.

**Technical indicators**	
Passes	An intentional disposal of the ball with the aim to be received by a teammate.
Accurate passes (%)	The percentage of passes which were actually received by a team.
Shots	An attempt at goal, with the intention to score, made with any (legal) part of the body.
Crosses	Any ball played into the opposition team’s penalty area from a wide position.
Dribbles	An attempt by a player in possession of the ball, to evade or move past an opponent while still in control of the ball.
Air challenges	Two players contesting for an aerial ball.
Air challenges won	A player who wins an aerial ball after the contest between two players.
Tackles	The act of gaining possession from an opposition player, when they are in possession of the ball.
Tackles won (%)	The proportion of successful tackles, whereby a player removes the opposition from possession of the ball and possession is retained by either themselves or one of their teammates.
Lost balls	Loss of ball possession due to a mistake/poor control.
Fouls	Any infringement that is penalised as foul play by a referee.

**Table 2:** Physical and technical indicators according to playing position

	Central Defenders (n = 45)	Wide Defenders (n = 46)	Central Midfielders (n = 50)	Wide Midfielders (n = 17)	Forwards (n = 22)
**Physical indicators**					
Walking (m)	3761 ± 252	3743 ± 232	3531 ± 254	3801 ± 300	3885 ± 409
Jogging (m)	3620 ± 540	3792 ± 467	4249 ± 445	3911 ± 583	3405 ± 753
Running (m)	1281 ± 268	1532 ± 287	1942 ± 414	1668 ± 376	1324 ± 330
High-speed running (m)	498 ± 137	710 ± 159	739 ± 202	796 ± 202	631 ± 165
Sprinting (m)	67 ± 42	152 ± 199	91 ± 66	214 ± 170	138 ± 94
Total distance (m)	9226 ± 720	9929 ± 633	10553 ± 763*	10390 ± 844	9383 ± 820

**Technical indicators**					
Passes	44.69 ± 19.28	49.67 ± 17.92	52.36 ± 15.12#	36.71 ± 13.22	28.23 ± 9.78
Accurate passes (%)	86 ± 9	84 ± 7	8 ± 5#	79 ± 7	7 ± 9
Shots	0.31 ± 0.60	0.30 ± 0.51	0.98 ± 1.22	1.41 ± 1.23	2.23 ± 1.57
Crosses	0.00 ± 0.00	2.07 ± 2.02$	0.38 ± 0.57	1.06 ± 1.14	0.68 ± 0.78
Dribbles	0.31 ± 0.60	2.00 ± 1.73	2.06 ± 1.99	3.71 ± 2.66	4.50 ± 3.02¥
Air challenges	4.78 ± 2.83	3.00 ± 2.01	3.80 ± 3.89	3.65 ± 3.33	7.64 ± 6.37
Air challenges won	3.27 ± 1.98	1.43 ± 1.17	2.08 ± 2.10	1.59 ± 2.50	3.00 ± 3.15
Tackles	2.51 ± 2.17	3.74 ± 2.27	4.74 ± 2.86	2.94 ± 1.68	2.09 ± 1.63
Tackles won (%)	58 ± 40	63 ± 29	5 ± 28	56 ± 32	31 ± 34
Lost balls	2.67 ± 1.80	5.09 ± 2.66	5.02 ± 2.58	7.76 ± 3.42	10.55 ± 5.60&
Fouls	0.91 ± 0.97	1.48 ± 1.09	1.94 ± 1.52€	1.35 ± 1.27	1.73 ± 1.35

Data are expressed as mean ± SD. Unless a unit is provided, data are presented as counts. The following indicate significance (p<0.05):

* Significantly higher than Central Defenders, Wide Defenders and Forwards;

# Significantly higher than Wide Midfielders and Forwards;

$ Significantly higher than Wide Midfielders;

& Significantly higher than all playing positions;

¥ Significantly higher than Central Defenders, Wide Defenders and Central Midfielders;

€ Significantly higher than Central Defenders.
